# An Analytically Solvable Model for Rapid Evolution of Modular Structure

**DOI:** 10.1371/journal.pcbi.1000355

**Published:** 2009-04-10

**Authors:** Nadav Kashtan, Avi E. Mayo, Tomer Kalisky, Uri Alon

**Affiliations:** 1 Department of Molecular Cell Biology, Weizmann Institute of Science, Rehovot, Israel; 2 Physics of Complex Systems, Weizmann Institute of Science, Rehovot, Israel; University of Washington, United States of America

## Abstract

Biological systems often display modularity, in the sense that they can be decomposed into nearly independent subsystems. Recent studies have suggested that modular structure can spontaneously emerge if goals (environments) change over time, such that each new goal shares the same set of sub-problems with previous goals. Such modularly varying goals can also dramatically speed up evolution, relative to evolution under a constant goal. These studies were based on simulations of model systems, such as logic circuits and RNA structure, which are generally not easy to treat analytically. We present, here, a simple model for evolution under modularly varying goals that can be solved analytically. This model helps to understand some of the fundamental mechanisms that lead to rapid emergence of modular structure under modularly varying goals. In particular, the model suggests a mechanism for the dramatic speedup in evolution observed under such temporally varying goals.

## Author Summary

Biological systems often display modularity, in the sense that they can be decomposed into nearly independent subsystems. The evolutionary origin of modularity has recently been the focus of renewed attention. A series of studies suggested that modularity can spontaneously emerge in environments that vary over time in a modular fashion—goals composed of the same set of subgoals but each time in a different combination. In addition to spontaneous generation of modularity, evolution was found to be dramatically accelerated under such varying environments. The time to achieve a given goal was much shorter under varying environments in comparison to constant conditions. These studies were based on computer simulations of simple model systems such as logic circuits and RNA secondary structure. Here, we take this a step forward. We present a simple mathematical model that can be solved analytically and suggests mechanisms that lead to the rapid emergence of modular structure.

## Introduction

Biological systems often display modularity, defined as the seperability of the design into units that perform independently, at least to a first approximation [1]–[5]. Modularity can be seen in the design of organisms (organs, limbs, sensory systems), in the design of regulatory networks in the cell (signaling pathways, transcription modules) and even in the design of many bio-molecules (protein domains).

The evolution of modularity has been a puzzle because computer simulations of evolution are well-known to lead to non-modular solutions. This tendency of simulations to evolve non-modular structures is familiar in fields such as evolution of neural networks, evolution of hardware and evolution of software. In almost all cases, the evolved systems cannot be decomposed into sub-systems, and are difficult to understand intuitively [6]. Non-modular solutions are found because they are far more numerous than modular designs, and are usually more optimal. Even if a modular solution is provided as an initial condition, evolution in simulations rapidly moves towards non-modular solutions. This loss of modularity occurs because there are so many changes that reduce modularity, by forming connections between modules, that almost always a change is found that increases fitness.

**Figure 1:**
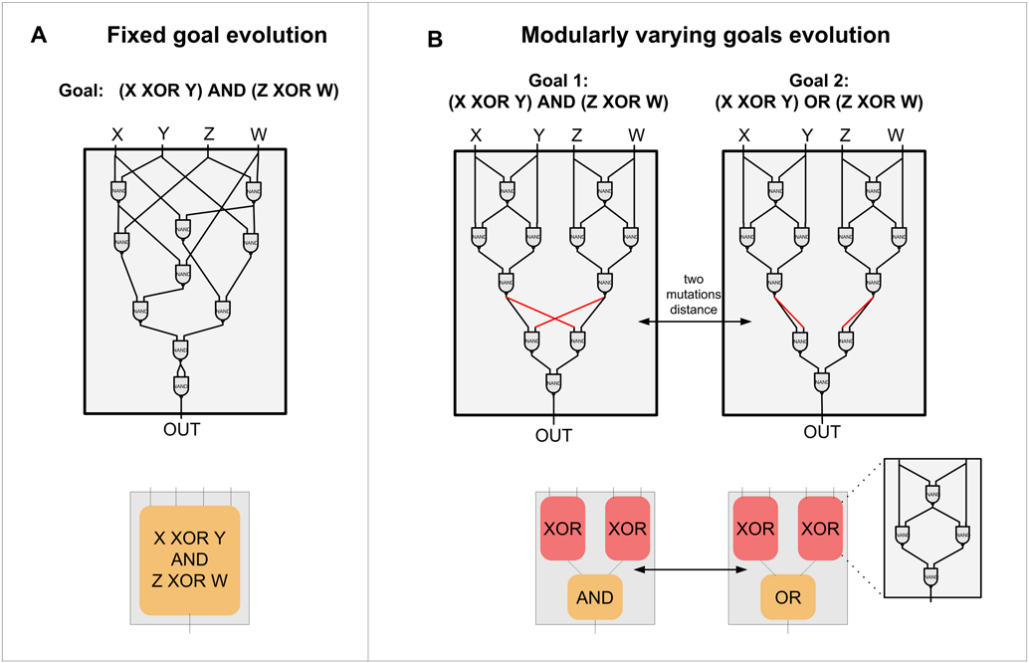
Evolution under Fixed Goals (FG) and Modularly Varying Goals (MVG). Examples of data from a series of studies [18],[19] that suggest that modularity spontaneously evolves when goals change over time in a modular fashion (modularly varying goals or MVG). (A) Logic circuits made of NAND gates evolved under a constant goal (fixed goal, abbreviated FG) that does not vary over time, *G*_1_ = (x XOR y) AND (w XOR z). The circuit is composed of 10 NAND gates. Evolution under a constant goal typically yields compact non-modular circuits. (B) Circuits evolved under MVG evolution, varying every 20 generations between goal *G*_1_ and goal *G*_2_ = (x XOR y) OR (w XOR z). Note that these two goals share the same sub-goals, namely two XOR functions. Connections that are rewired when the goal switches are marked in red. Evolution under MVG typically yields modular circuits that are less compact, composed in this case of 11 gates. The circuits are composed of three modules: two XOR modules and a third module that implements an AND/OR function, depending on the goal.

Several suggestions have been made to address the origin of modularity in biological evolution [5], [7]–[16], recently reviewed by Wagner et al [17]. Here we focus on a recent series of studies that demonstrated the spontaneous evolution of modular structure when goals vary over time. These studies used computer simulations of a range of systems including logic circuits, neural networks and RNA secondary structure. They showed that modular structures spontaneously arise if goals vary over time, such that each new goal shares the same set of sub-problems with previous goals [18]. This scenario is called *modularly varying goals*, or MVG. Under MVG, modules spontaneously evolve. Each module corresponds to one of the sub-goals shared by the different varying goals. When goals change, mutations that rewire these modules are rapidly fixed in the population to adapt to the new goal (Figure 1 A,B).

**Figure 2:**
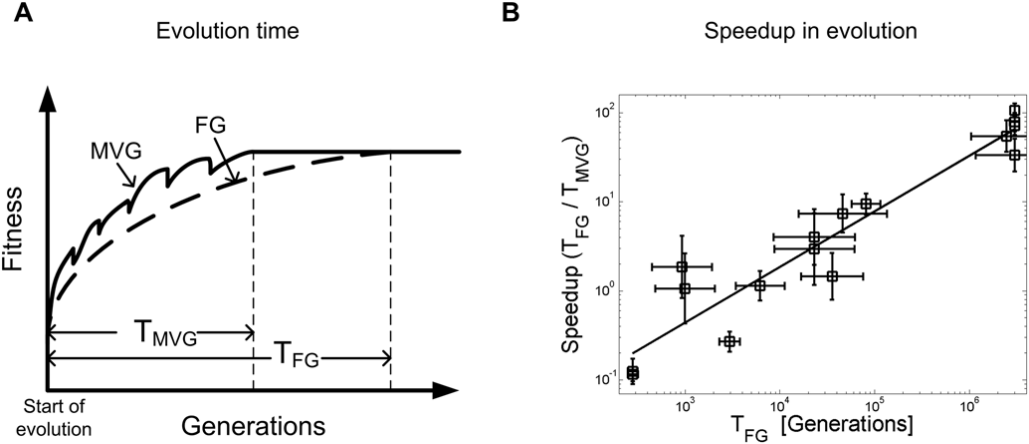
Speedup of evolution under MVG. (A) A schematic view of fitness as a function of generations in evolution under MVG and fixed (constant) goal (FG). Evolution time (*T_MVG_* and *T_FG_*) is defined as the median number of generations it takes to achieve the goal (i.e. reach a perfect solution) starting from random initial genomes. (B) Speedup of evolution under MVG based on simulations of logic circuits with goals of increasing complexity (see [19]). The speedup is defined as evolution time under a fixed goal, divided by evolution time under MVG that switches between the same goal and other modularly related goals: *S = T_FG_/T_MVG_*. Shown is the speedup *S* versus the evolution time under fixed goal (*T_FG_*). Speedup scales approximately as a power law *S∼(T_FG_) ^α^* with an exponent α = 0.7±0.1. Thus, the harder the goal the larger the speedup.

In addition to promoting modularity, MVG was also found to dramatically speed evolution relative to evolution under a constant goal [19]. MVG speeds evolution in the sense that it reduces the number of generations needed to achieve the goal, starting from initial random genomes. Despite the fact that goals change over time, a situation that might be thought to confuse the evolutionary search, the convergence to the solution is much faster than in the case of a constant goal (Figure 2 A,B). Intriguingly, the harder the goal, the faster the speedup afforded by MVG evolution.

To summarize the main findings of [18],[19]:

- A constant goal (that does not change over time) leads to non-modular structures.
- Modularly varying goals lead to modular structures.
- Evolution converges under MVG much faster than under a constant goal.
- The harder the goals, the faster the speedup observed in MVG relative to constant goal evolution.
- Random (non-modular) goals that vary over time usually lead to evolutionary confusion without generating modular structure, and rarely lead to speedup.

Since these findings were based on simulations, it is of interest to try to find a model that can be solved analytically so that the reasons for the emergence of modular structure, and for the speedup of evolution, can be more fully understood. Here we present such a simple, exactly solvable model. The model allows one to understand some of the mechanisms that lead to modularity and speedup in evolution.

## Model

### Definition of the system

The guiding principle in building the model was to find the simplest system that shows the salient features described in the introduction. It turns out that many of these features can be studied using a linear system, similar to those used in previous theoretical work on evolution [8], [20]–[23]. Consider a system that provides an output for each given input. The input is a vector of *N* numbers. For example, the input can represent the abundance of *N* different resources in the environment. The output is also a vector of *N* numbers, for example the expression of the genes that utilize the resources. The structure that evolves is represented by an *N*×*N* matrix, **A**, that transforms the input vector **v** to a desired output vector **u** such that:
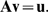
(1)


The matrix **A** can be thought of, quite generally, as the linearized response of a biological regulatory system that maps inputs to outputs, taken near a steady-state of the system. In this case the vectors **u** and **v** represent perturbations around a mean level, and can have negative or positive elements.

### Goals are desired input-output relations

An evolutionary goal in the present study is that an input vector **v** gives a certain output vector **u**. We will generally consider goals *G* that are composed of *k* such input-output vector pairs.

### The fitness is the benefit minus the cost of matrix elements

To evaluate the fitness of the system, we follow experimental studies in bacteria, that suggest that biological circuits can be assigned benefit and cost [24]. The benefit is the increase in fitness due to the proper function of the circuit, and the cost is the decrease in fitness due to the burden of producing and maintaining the circuit elements. In this framework, fitness is the benefit minus the cost of a given structure **A**.

We begin with the cost of the system, related to the magnitude of the elements of **A**. We use a cost proportional to the sum over the squares of all the elements of **A**:
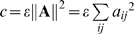
. This cost represents the reduction in fitness due to the need to produce the system elements. A quadratic cost function resembles the cost of protein production in *E. coli* [24]–[26]. The cost tends to make the elements of **A** as small as possible. Other forms for the cost function, including sum of absolute values of *a_ij_* and saturating functions of *a_ij_*, are found to give similar conclusions as the quadratic cost function (see Text S1).

In addition to the cost, each structure has a benefit. The benefit *b* of a structure **A** is higher the closer the actual output is to the desired output: 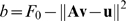 (where *F_o_* represents the maximal benefit). In the case where the goal includes *k* input-output pairs, one can arrange all input vectors in a matrix **V**, and all output vectors in a matrix **U**, and the benefit is the sum over the distances between the actual outputs and the desired outputs 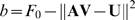. In total, the fitness of **A** is the benefit minus the cost:
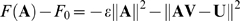
(2)


The first term on the right hand side represents the cost of the elements of **A**, and the second term is the benefit based on the distance between the actual output, **AV**, and the desired output, **U**. The parameter *ε* sets the relative importance of the first term relative to the second.

In realistic situations, the parameter *ε* is relatively small, because getting the correct output is more important for fitness than minimizing the elements of **A**. Thus, throughout, we will work in the limit of *ε* much smaller than the typical values of the elements of the input-output vectors.

Now that we have defined the fitness function, we turn to the definition of modularity in structures and in goals.

### Definition of modularity

**Figure 3:**
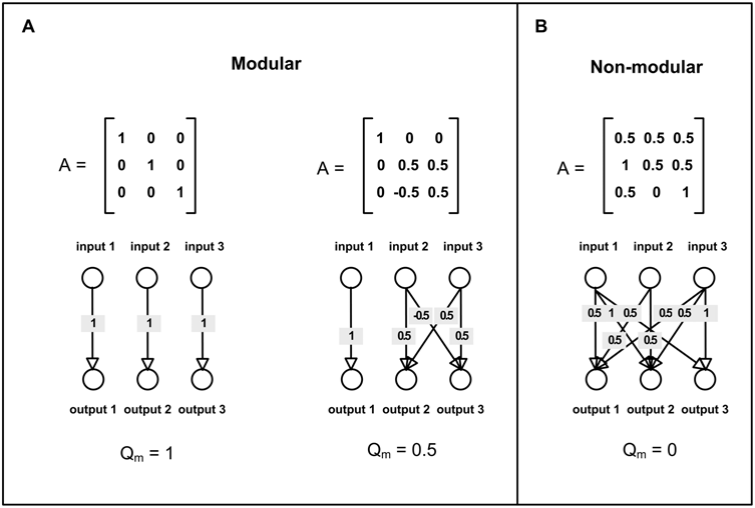
Modularity of matrices and their corresponding networks. The *NxN* matrix **A** can be represented as a directed network of weighted interactions between the inputs and the outputs (with 2*N* nodes). Modularity is measured using normalized measure of community structure of the interaction network, *Q_m_* (see Text S1) [18]. (A) Examples of two modular matrices and their corresponding modularity measure *Q_m_*. Modular matrices typically show *Q_m_*>0.2, with a maximal value of *Q_m_* = 1 for a diagonal matrix. (B) An example of a non-modular matrix. Non-modular matrices have *Q_m_* around 0.

A modular structure, which corresponds to a modular matrix **A**, is simply a matrix with a block diagonal form (Figure 3). Such matrices have non-zero elements in blocks around the diagonal, and zero elements everywhere else. Each block on the diagonal maps a group of input vector components to the corresponding group of output vector components. An example of a modular structure is
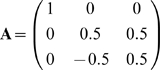



In addition to the modularity of the structure **A**, one needs to define the modularity of varying goals. In the present study, modularity of a goal is defined as the ability to separate the input and output components of **u** and **v** into two or more groups, such that the outputs in each groups are a function only of the inputs in that group, and not on the inputs in other groups. Thus, the inputs and outputs in a modular goal are separable into modules, which can be considered independently (Figure 3). In the present linear model we require that the outputs in each group are a linear function of the inputs in that group. For example, consider the following goal *G_o_* that is made of two input-output pairs:
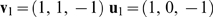
and
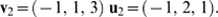



Here the first component of each output vector is a linear function of the first component of the corresponding input vectors, namely the identity function. The next two components of each output vector are equal to a linear 2×2 matrix, **L** = [(0.5,0.5);(−0.5, 0.5)], times the same two components of the input vector. In fact, the modular matrix **A** given above satisfies this goal, since **Av_1_ = u_1_** and **Av_2_ = u_2_**. Thus, the input-output vectors in *G*_o_ can be decomposed into independent groups of components, using the same linear functions. Hence, the goal *G*_o_ is modular. Note that most goals (most input-output vector sets with *N*>2) cannot be so decomposed, and are thus non-modular.

To quantify the modularity of a structure **A** we used the modularity measure *Q_m_* based on the Newman and Girvan measure [18],[27], described in [18] and also in the Text S1. Under this measure, diagonal matrices have high modularity, block modular matrices show intermediate modularity and matrices with non-zero elements that are uniformly spread over the matrix have modularity close to zero (Figure 3).

## Results

In the following sections we analyze the dynamics and convergence of evolution under both fixed goal conditions and under MVG conditions. For clarity we first present a two–dimensional system (*N* = 2), and then move to present the general case of high-dimension systems. Each of the sections is accompanied by detailed examples that are given to help to understand the system behavior. The third section describes full analytic solutions and proofs.

### Evolution dynamics and convergence in two-dimensions

#### A constant goal generally leads to a non-modular structure

We begin with two-dimensional system (*N* = 2), so that **A** is a two by two matrix. We note that the two-dimensional case is a degenerate case of MVG, but has the advantage of easy visualization. It thus can serves as an introduction to the more general case of higher dimensions, to which we will turn later.

Consider the goal *G_1_* defined by the input vector **v** = (1, 1) and its desired output **u** = (1, 1). Note that in the case of *N* = 2 all goals are modular according to the above definition (because there exists a diagonal matrix that satisfies **Av** = **u**). In the case of goal *G_1_*, the identity matrix **A** = [(1, 0), (0, 1)] satisfies the goal.

Let us find the most fit structure **A**, given the goal *G_1_*. To find the structure **A** that maximizes the fitness *F*(**A**), we ask when the matrix elements of **A**, *a_ij_*, satisfy 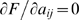. From Eq. 2, this leads to the following 4 equations, one for each of the 4 elements of **A**:
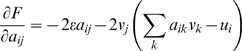
(3)


Solving these equations, we find that the highest fitness structure is 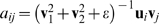. Upon substituting **v** and **u** we get:
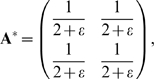
and when the cost is small (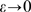) one has
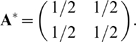



Note that indeed, **A**·**v** = **u**, so that the goal is satisfied. Thus, *the optimal solution is non modular*. This non-modular matrix satisfies the goal and also keeps the elements of the matrix small to minimize the cost (see section *Full analytic solutions (A)*). The modular solution,
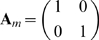
is less fit because of the higher cost of its elements: the cost is proportional to the sum of the squares of the elements, so that the cost for the modular matrix **A_m_**, *c = 2ε* , is higher than the cost for the highest-fitness matrix A^*^, *c = ε*.

**Figure 4:**
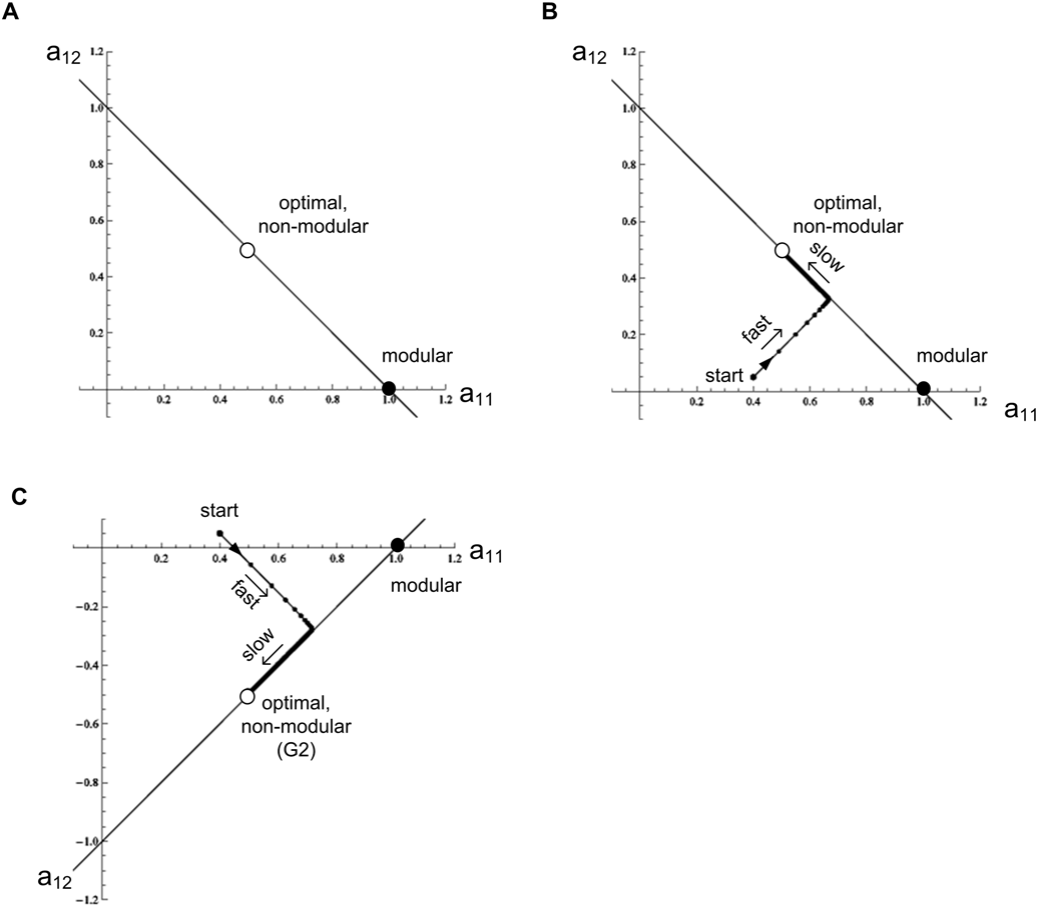
Dynamics of evolution under a constant goal. (A) Matrix elements are portrayed in a two dimensional space defined by *a_11_* and *a_12_*, the first row elements of the matrix **A**. The goal is *G_1_* = [**v** = (1,1), **u** = (1,1)], empty circle: optimal non-modular solution (0.5, 0.5). Full circle: modular solution (1,0). The line *a_12_* = 1−*a_11_* represents all configurations that satisfy the goal (satisfy *Av* *=* *u*). (B) A typical trajectory under the constant goal *G*_1_. Black dots display the dynamics at 100/r time unit resolution, where *r* is the rate in Eq. 4. (C) Same as (B) for the goal *G_2_* = [**v** = (1,−1), **u** = (1,−1)].

It is also helpful to graphically display this solution. Figure 4A shows the two-dimensional space defined by the first row of **A**, the elements *a_11_* and *a_12_*. The matrices **A** that satisfy the goal (give **Av = u**) correspond to a line, *a_11_+a_12_* = 1. The modular solution is the point that intersects the axes at *a_11_* = 1,*a_12_* = 0. The optimal solution A^*^ is at the point (*a_11_, a_12_*) = (*1/2, 1/2*).

A non-modular solution is the general solution for this type of goal (proof in section *Full analytic solutions (A)*). For the benefit of the next section, we consider briefly a second example, the goal *G_2_*, **v** = (1, −1), **u** = (1, −1). As in the case of *G*_1_, the highest-fitness structure for G_2_ is non-modular, (Figure 4C)
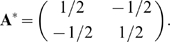



#### Convergence is slow under a constant goal

We now turn to discuss the dynamics of the evolutionary process. We ask how long it takes to reach the maximum-fitness structure starting from a random initial structure. For this purpose, one needs to define the dynamics of evolutionary change and selection. For simplicity, we consider a Hill-climbing picture, in which the rate of change of the structure **A** is proportional to the slope of the fitness function. The rate of change is high along directions with high fitness gradients and slow along directions with small gradients. Thus 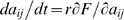 where *r* is the ‘rate’ of evolution, based on the rate at which mutations that change *a_ij_* occur and are fixed in the population. We note that similar results are found when using genetic algorithms with more realistic mutation and selection strategies (see Text S1).

The Hill-climbing dynamical model is simple enough to analytically solve for the dynamics of the matrix elements *a_ij_*. For a constant goal, one has (with time rescaled to take the evolution rate into account, 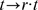):
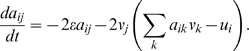
(4)


These are linear ordinary differential equations, and hence the solution for *a_ij_* is of the form:
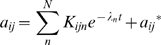
(5)where {λ*_n_*} are the eigenvalues of Eq. 4. The prefactors {*K_n_*} are determined by the eigenvectors corresponding to {*λ_n_*}, and the initial conditions. The structures converge to 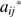, which is the value of the matrix elements *a_ij_* in the optimal solution.

The convergence times are thus governed by the eignevalues *λ_n_*. In particular, the smallest eigenvalue corresponds to the longest convergence time. We find that in the case of a constant goal that does not vary with time, the smallest eigenvalue is always equal to 2*ε* (for a proof see results section *Full analytic solutions (B)*).

For example, for the goal *G_1_*, the four eigenvalues of Eq. 4 are *λ*_1_ = *λ*_2_ = *2ε* and *λ*_3_ = *λ*_4_ = *2*(*2+ε*). The large eigenvalues *λ*_3_ and *λ*_4_ correspond to rapid evolution to the line shown in Figure 4B. The small eigenvalues *λ*_1_ = *λ*_2_ = *2ε* correspond to motion along the line, converging as *exp*(*−λ_1_ t*) to the optimal solution. Thus, the convergence time for small *ε* is very long, *T_FG_∼1/λ_1_∼1/ε* (‘FG’ stands for fixed goal). The same applies to the goal *G_2_*, in which the two small eigenvalues *λ*_1_ = *λ*_2_ = *2ε* govern the slow motion along the line on Figure 4C. The lines in Figure 4B and Figure 4C along which evolution moves slowly are analogous to the fitness plateaus or neutral networks observed in the evolution of more complex systems [28]–[31].

#### Varying between modular goals leads to modular structure

We next consider the case where the environment changes over time, switching between the two modular goals mentioned above. For example, the structure **A** evolves towards goal *G_1_*, defined by **v_1_** = (1, 1) and **u_1_** = (1, 1). Then, the goal changes to a different goal *G_2_*, defined by **v_2_** = (1, −1) and **u_2_** = (1, −1). After some time, the goal returns to the first goal, and so on. The goals thus switch from time to time from *G_1_* to *G_2_* and back. Looking at these two goals, it is seen that each component of the output vectors can be determined only by the corresponding component in the input vector. Another way to say this is that the same modular matrix **A** = [(1,0),(0,1)], satisfies both *G_1_* and *G_2_*. This is thus an example of modularly varying goals, or MVG for short.

What is the structure that evolves under MVG? We use the dynamical equations (Eq. 4) to describe the MVG process which switches between the goals.
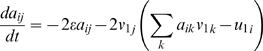
(6a)

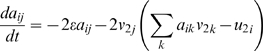
(6b)


**Figure 5:**
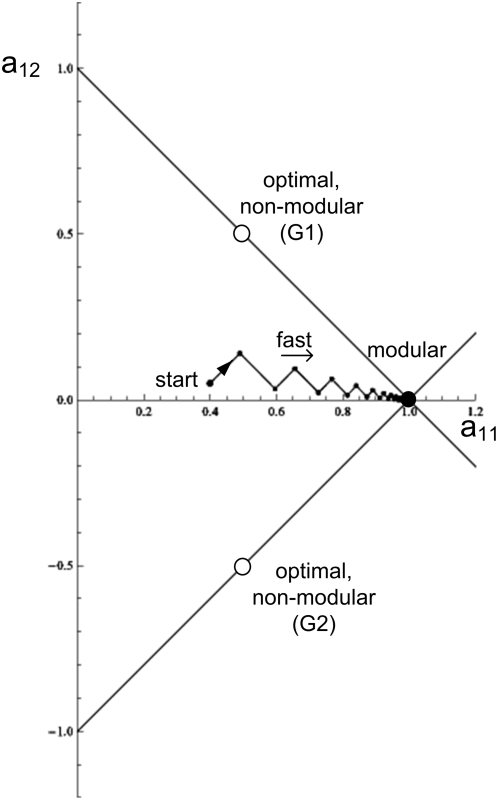
Evolutionary dynamics under modularly varying goals (MVG) converges to the modular solution. Goals are switched between *G*_1_ = [**v** = (1,1), **u** = (1,1)] and *G*_2_ = [**v** = (1,−1), **u** = (1,−1)] every *t = 100/r* time units. A typical trajectory of the matrix elements is shown, where small black dots represent the dynamics of the system in *100/r* time steps resolution. Empty and full circles represent the optimal and modular solutions respectively.

Here Eqs. 6a and 6b are valid for times when the goals are *G_1_* and *G_2_* respectively. One finds that the structure **A** evolves towards the modular solution **A** = [(1, 0);(0, 1)]. As shown in Figure 5, when the goal is equal to *G_1_*, the elements of **A** move towards the line of *G_1_* solutions, and when the goal changes to *G_2_*, the elements of **A** move towards the line of *G_2_* solutions. Together, these two motions move **A** towards the modular solution at which the two lines intersect (Figure 5).

To analyze this scenario, consider the limiting case where switches between the two goals occur very rapidly. In this case, one can average the fitness over time, and ask which structure maximizes the average fitness. If the environment spends, say, half of the time with goal *G_1_*, and half of the time with goal *G_2_*, then the average fitness is
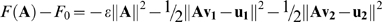
(7)


One can then solve the equations for the elements *a_ij_* of the matrix **A** that maximize fitness. The result is that the structure that optimizes fitness is the modular matrix **A** = [(*1,0*),(*0,1*)] (see section *Full analytic solutions (A)* for the general proof). This modular solution is found regardless of the fraction of time spent in each of the goals (as long as this fraction is not close to *1/ε*, in which case one returns to a constant-goal scenario).

Intuitively, supplying two modular goals provides ‘extra information’ that helps evolution find the unique structure that satisfies both goals – even though the different goals do not appear at the same time. If one stops varying the goals and presents a constant goal *G_1_* or *G_2_*, the structure evolves to the non-modular structures mentioned in previous sections. Thus, when the goals vary in time, the system ‘remembers’ the previous goal. This memory guides it towards the modular solution, even though at each time point, the current goal does not contain sufficient information to specify that solution.

#### Varying between modular goals speeds convergence to solution

We have seen that MVG leads to a modular structure. Let us now analyze the time that it takes the evolutionary process to approach this modular solution, starting from a random initial condition. In contrast to the small eigenvalues (long convergence time) found under a constant goal, a different situation is found under MVG. Here, evolution converges rapidly to the modular solution, with convergence time of order one *T_MVG_∼1*. In MVG, the small, order-*ε* eigenvalues are eliminated and all eigenvalues are generally large resulting in fast dynamics (see proof for the general case in the section *Full analytic solutions (B)*).

To understand why dynamics are rapid, consider the view depicted in Figure 5, showing the trajectories of **A** as the goal varies over time. One sees a rapid convergence to the line representing the current goal, and then, once the goal changes, a rapid move to the line representing the new goal. Thus, provided that switching is not very slow (that is when the switching time *E* are shorter than the time to solve under a constant goal: *E*<*T_FG_ ∼1/ε*), it is the large eigenvalues that governs the dynamics and lead to rapid convergence. Modularly varying goals cause evolution to converge rapidly on the modular solution. Similar results are found using genetic algorithms instead of Hill-climbing evolutionary equations (see Text S1).

**Figure 6:**
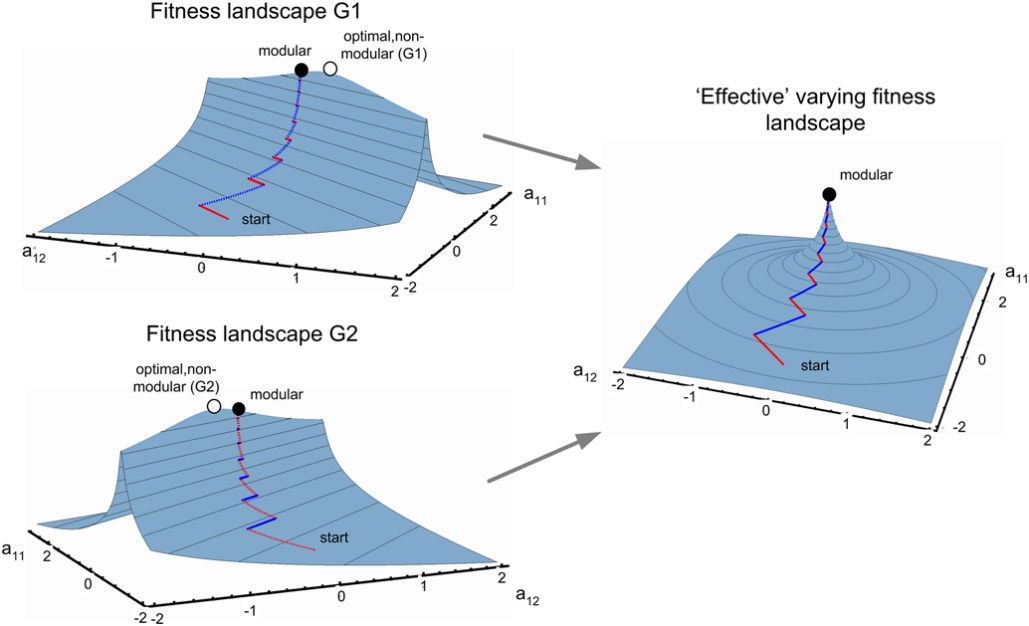
Fitness landscape illustration (a two dimensional system). Goals *G_1_*, *G_2_* are as described in Figures 4,5. The fitness landscapes are presented as a projection on the hyper-surfaces (*a_21_, a_22_*) of the optimal solution [i.e. (0.5,0.5) for *G_1_*, (−0.5, 0.5) for *G_2_*, and (0, 1) for MVG]. A typical trajectory is shown under MVG, switching between *G_1_* and *G_2_* as described in Figure 5. red/blue: dynamics under fitness landscape *G_1_* and *G_2_* respectively. Fitness is presented in log scale. Full/empty circle represents the modular/non-modular solutions. The fitness landscapes under constant goals are characterized by a single ridge (with slow dynamics as shown in Figures 4B and 4C). Under MVG the effective fitness landscape forms a steep peak where a solution that solves both goals resides (the modular solution). To ease comprehension, we chose a different viewing angle from the one of Figures 4,5. Switching time is *E* = 100/*r*.

It is also helpful to visually examine the fitness landscapes that govern the dynamics of MVG. One can get a feeling for the shape of the landscape by looking at the fitness function averaged over both goals. The rapid convergence to a modular solution is due to the formation of a steep peak in the ‘effective’ combined fitness landscape, as opposed to a flat ridge in the case of evolution under a constant goal (Figure 6).

### Evolution dynamics, convergence and modularity in higher dimensions

#### In higher dimensions, MVG also leads to a modular structure and speedup

The two dimensional case we have discussed is relatively easy to visualize. Let us now consider higher dimensions. We will consider a three-dimensional problem (*N = 3*), bearing in mind that the conclusions turn out to be valid for all dimensions 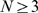. Here, each goal will be composed of *k* input-output pairs. In general evolutionary problems, involving systems such as logic gates, neuronal networks or RNA molecules, there are numerous different solutions to each goal. To mimic this, we keep the number of input-output pairs in each goal not too large, specifically *k = N−1*. This assures an infinite number of solutions to the goal (if 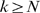, at most a single solution exists since the number of unknown matrix elements is smaller than the number of equations given by the *k* input-output pairs). In our *N = 3* example, each goal is thus be made of *k = 2* input-output vector pairs.

Let us begin with the goal *G_1_* which consists of the following pairs
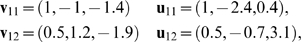



Note that *G_1_* is modular: the input-output vectors in *G_1_* can be decomposed into independent groups: the first component in the input is simply equal to the first component in the output, and the next two components are related to the output components by a linear transformation **L** = [(1,1);(1,−1)]. Hence, there exists a block-modular matrix **A** = [(1,0,0);(0,1,1);(0,1,−1)] that satisfies this goal. However, when *G_1_* is applied as a constant goal, the optimal solution (assuming 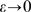) is non-modular (fitness = −3.7ε)
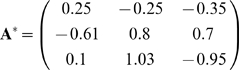



**Figure 7:**
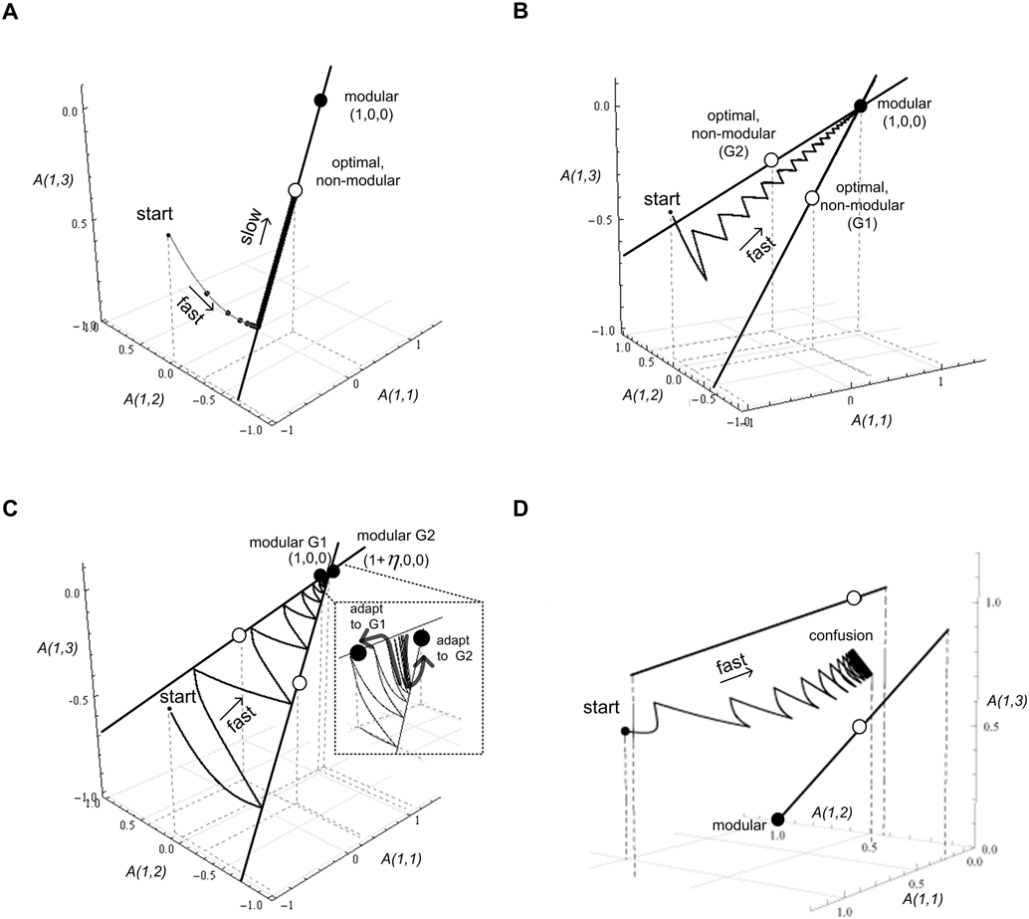
Dynamics on a 3-dimensional system (A = *3*×*3 matrix*). Presented is the three dimensional space defined by *a_11_*, *a_12_*, and *a_13_*, the first row elements of the matrix **A**. The goal is defined by two pairs of input-output vectors. Empty circle: optimal non-modular solutions. Full circle: modular solutions. A typical trajectory is shown for a number of different cases. Lines represent all configurations that achieve the goal (satisfy **Av**_**1**_ = **u**_**1**_ and **Av**_**2**_ = **u**_**2**_). (A) A Constant goal *G_1_* = { [**v**_11_ = (1,−1,−1.4), **u**_**11**_ = (1,−2.4,0.4)]; [**v**_**12**_ = (0.5,1.2,−1.9), **u**_**12**_ = (0.5,−0.7,3.1) ] }. (B) Modularly varying goals. *G*_1_ as above, and *G_2_* = { [ **v**_**11**_ = (1,1.7,−0.7), **u**_**11**_ = (1,1,2.4) ]; [ **v**_**12**_ = (−0.7,−2.3,−1.1), **u**_**12**_ = (−0.7,−3.4,−1.2) ] }. Switching rate is *E* = 100/*r* time steps. (C) Modularly varying goals with nearly identical modules: *G_1_* = { [ (1,1.7,−0.7), (1,1,2.4) ]; [ (−0.7,−2.3,−1.1), (−0.7,−3.4,−1.2) ] } and *G_2_* = { [ (1,−1,−1.4), (1+*η*,−2.4,0.4) ]; [ (0.5,1.2,−1.9), (0.5+0.5*η*,−0.7,3.1) ] }. The distance between the two modular solutions for each of the goals is *η = 0.1*. Zoom in: adaptation dynamics between the modular solutions. (D) Random non-modular varying goals: *G_1_* = { [ (−2.5,1,1), (0,1,1) ]; [ (5.4,−1,1), (3,−1,1) ] }, G_2_ = { [ (1.1,1,1), (1.1,1,1) ]; [ (0.6,−1,1), (0.6,−1,1) ] }. *E = 100/r* time steps. There is no solution that solves both goals well, and therefore the dynamics lead to ‘confusion’, a situation where none of the goals are achieved.

The dynamical equations have a small eigenvalue *λ = 2ε*. Hence, convergence is slow, and takes *T_FG_∼1/ε*. The evolutionary process converges slowly along the line shown in Figure 7A, reaching the non-modular optimal structure.

**Figure 8:**
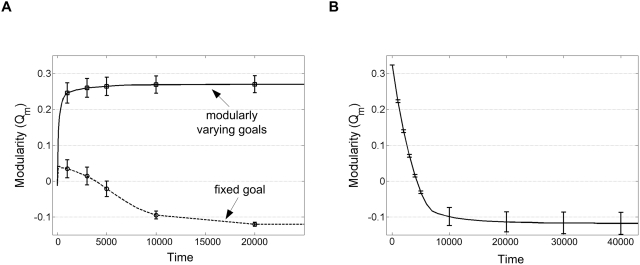
Modularity rises under MVG, and drops when goals stop varying over time. Modularity of the system measured by normalized community structure *Q_m_* (see Text S1). (A) MVG and FG scenarios. Mean±SE is of 20 different goals each with 20 different random initial conditions; *E = 10/r* (B) Starting from initial modular matrix **A** = [(1,0,0);(0,1,1);(0,1,−1)] evolved under MVG, at time *t = 0* the goals stopped changing (i.e. evolution under FG conditions from time *t = 0*). Mean±SE is of 20 different goals.

In contrast, if MVG is applied, switching between *G_1_* and a second goal *G_2_*, which share the same modular structure, say
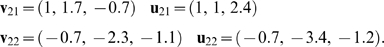
one finds a rapid convergence to a modular structure (Figure 7B; with fitness = −5*ε*):
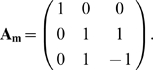



Modularity increases rapidly as shown in Figure 8A. These results are similar to the ones discussed in the *N* = 2 case of the previous section. Generalizing the results shows that MVG produces modular structures in any dimension, as shown below in the section *Full analytic solutions (A)*.

#### MVG with nearly identical modules

Up to now, the varying goals shared the same modular solution. Let us consider a more general case where the varying goals *G_1_* and *G_2_* have similar, but different, modular solutions. Specifically, the two goals share the same general modular structure but with slightly different modules. They can thus be solved by the same block matrix except for corrections on the order of a small parameter *η*. This situation is more similar, in a sense, to our previous simulations on complex model systems where each of the varying goals was solved by a different modular structure.

As an example, which represents the typical case, let *G_1_* = { [ (1, 1.7, −0.7), (1, 1, 2.4) ]; [ (−0.7, −2.3, −1.1), (−0.7, −3.4, −1.2) ] } which can be satisfied by the modular matrix
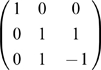
and *G_2_* = { [ (1, −1, −1.4), (1+*η*, −2.4, 0.4) ]; [ (0.5, 1.2, −1.9), (0.5(1+*η*), −0.7, 3.1) ] } which can be satisfied by the slightly different modular matrix
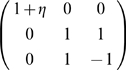



**Figure 9:**
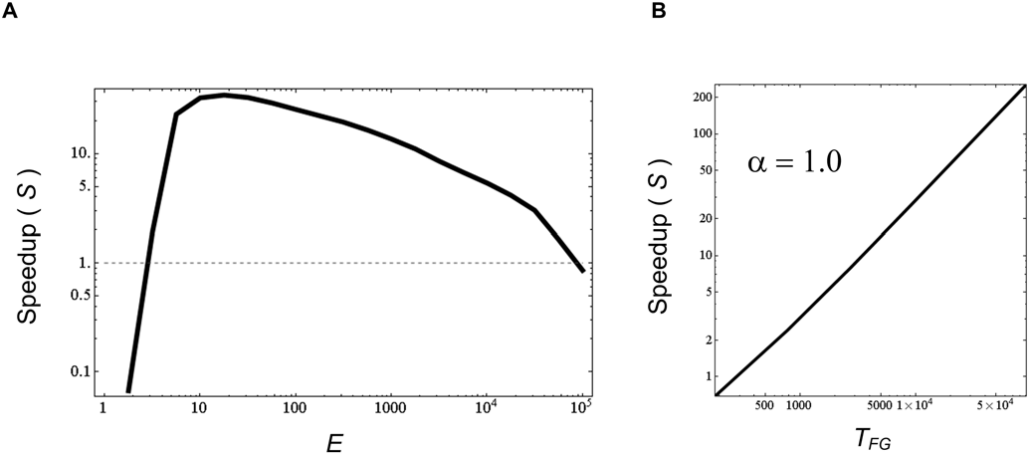
Evolution Speedup. (A) Speedup as a function of goal switching times *E*. Speedup is presented for the goal *G_1_* with MVG between the nearly modular pair of goals *G_1_* and *G_2_ : G_1_* = { [ (−0.4,−1.6,0.7), (−0.4,−1,−2.3) ]; [ (0,0.9,−0.3), (0,0.7,1.2) ] }, *G_2_* = { [ (2,−1.9,1.7), (2.9,−0.3,−3.6) ];[ (0.3,0.3,0.3), (0.4,0.6,−0.1) ] }, 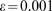. High speedup *S* is found for a wide range of goal switching times. (B) Speedup under MVG is greater the harder the goal (the more time it takes to solve the goal in FG evolution starting from random initial conditions). The Speedup *S = T_FG_ / T_MVG_* as a function of goal complexity, defined as the time to solve the goal under fixed goal evolution, *T_FG_*. The speedup scales linearly with *T_FG_*. Goals are as in (a). 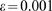 and *E = 10/r*.

We find that evolution under varying goals in such cases rapidly leads to a structure that is modular. Once the modular structure was established, the system moves between the two similar modular matrices every time the goal switches (Figure 7C). The degree of adaptation depends on the switching time between the goals: nearly perfect adaptation occurs when the switching time is large enough to allow the matrix elements to reach the modular matrix relevant for the current goal (roughly, switching that is slower than *η*/r, the ratio between distance between matrices *η* and the evolution rate *r*) (Figure 9A). Such cases suggest that evolved modular structure, although sub-optimal, is selected for the ability to adapt rapidly when the goal switches.

What is the effect of switching time (rate at which goals are switched) on the speedup? We find that speedup is high over a wide range of switching times. Speedup occurs provided that the switching times *E* are shorter than the time to solve under a constant goal (that is 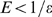). When switching times are long, the system behaves as if under a constant goal (for details see Text S1).

In the case of nearly-modular varying goals, speedup occurs provided that epoch times *E* are also long enough to allow evolution to adapt to the close-by modular solutions of the two goals (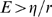 where *r* is the rate of evolution), but not too long, to avoid a crawl to the optimal solution (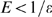) (Figure 9A).

#### Evolution of block-modular structures in higher dimensions

We briefly consider also a higher dimensional example with *N* = 6 and two goals, each composed of two input-output vectors as follows (values are rounded): *G_1_* is
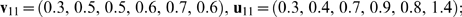


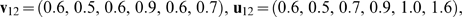
and *G_2_* is
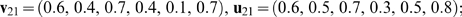


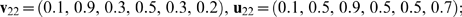



MVG evolution with these two goals converges to a block-modular structure
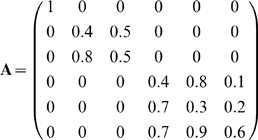



At this point, it is interesting to note that, in all dimensions, the block structure of the evolved matrix relates to the correlations within the goal input and output vectors. In fact, the block structure of **A** (the size and position of the blocks, not the value of each element) is the same as the block structure of the pair-wise linear correlation matrix between the goal inputs and outputs pairs (e.g. the correlation between the ‘input’ matrix whose rows are **v_11_, v_12_, v_21_, v_22_** to the ‘output’ matrix whose rows are **u_11_, u_12_, u_21_, u_22_** in the present example). A general proof is given in Text S1.

#### Modularity declines if goals become constant

What happens to modularity under a constant goal if one begins with a modular solution as an initial condition? We find that modularity decays over time (Figure 8B) with a typical time constant of 1/ε. Generally, this decay corresponds to motion along the low-gradient ridge towards the non-modular fixed point. (For a proof in *N* = 2 see Text S1). Thus, goals need to keep varying over time in order to maintain the modular structure.

#### Varying between random goals typically leads to evolutionary confusion

So far, we have considered modularly varying goals - that is goals that have a special feature: their components can be decomposed into modules, with the same (or nearly the same) modules for all goals. Thus, there exists a modular matrix **A** that satisfies (or nearly satisfies) all of the varying goals. What about randomly chosen goals, which are not modular in this sense?

Pairs of randomly chosen goals (with *N>2, k = N−1*) do not generally have a matrix **A** that satisfies both goals. Solving the dynamics in this case shows that temporally switching between the goals leads to confusion, where evolution does not find a good solution to either goal (Figure 7D).

It is easy to understand this using a geometrical picture. One can represent the set of solutions for each goal as a line (or hyper-plane) in the space of matrix elements. The solution lines of two random goals in the high dimension space have very low probability to cross or even to come close to each other. Switching between goals generally leads to a motion around the point where the lines come closest, which is generally a rather poor solution for each of the goals (Figure 7D).

Such confusion is avoided in the case of MVG, because goals share the same (or nearly the same) modular structure. Such a set of modular goals is special: it ensures that the corresponding lines intersect (or nearly intersect), and in particular that they intersect on one of the axes. One can prove (see section *Full analytic solutions (B)*), that all eigenvalues are of order one in the case of *g* modular goals each made of *k* input-output vectors (with *gk≥N* , so that sufficient information is available in the goals to specify a unique solution). Thus, in any dimension, a modular structure is rapidly found under MVG evolution.

There are special cases in which the goals are non-modular but still afford a speedup in evolution. This happens when the goal vectors happen to be linearly dependent such that a non-modular structure **A** exists that satisfies all goals. Here, rapid convergence under varying goals is found towards a non-modular structure. Geometrically, the hyper-planes corresponding to the goals happen to intersect at a point which is off the axes. This may correspond to the finding that randomly varying goals sometimes show mild speedup in simulations of complex models [19].

#### Speedup is greater the harder the goal

One can define the *speedup* of MVG compared to a constant goal, as the ratio of the convergence time under a constant goal to the convergence time in an MVG scenario [19],
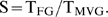
(8)


As pointed out above, the convergence time in a fixed goal (with dynamics mostly *along* the low-gradient lines) is determined by the small eigenvalues on the order of *ε*. Hence, *T_FG_∼1/ε*. In contrast, the convergence time in a modularly varying goal is determined by the larger eigenvalues *λ* which are order 1. Hence, *T_MVG_∼1/λ*, and
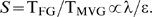
(9)


Thus, the ‘harder’ the fixed goal problem is (that is, the smaller *ε* and hence the longer *T_FG_*), the greater the speedup afforded by MVG (Figure 9B). A similar finding was made by simulations of the complex models such as logic circuits and RNA structures, which displayed *S*∝*(T_FG_)^α^* with *α* between 0.7 and 1.0 (Figure 2B) [19].

### Full analytic solutions

#### (A) The optimal solution in a fixed goal (FG) and in modularly varying goals (MVG)

Here we calculate the optimal solution in a problem in which the goal is fixed (FG), and in a problem with modularly varying goals (MVG). We show that the fitness of the optimal solution in a FG problem is higher than the fitness of the solution in a MVG problem.

We begin by considering the fitness function of Eq. 2 written in matrix form.
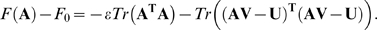
(A1)


Here **A** is an *N*×*N* matrix. **V** and **U** are both *N*×*k* matrices whose columns corresponds to the goal input vectors and output vectors *G*. The goal *G* is modular if there exists a block diagonal matrix **M** such that **MV = U** (up to permutations of the columns of **V** and **U**). We assume that the *k* columns of **V** are linearly independent. Note that if *k* = *N* then **V** is invertible and so 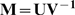. In the present study *k<N* so that there exist infinite number of matrices 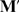 such that 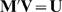.

The equation of motion for **A** in matrix notation (Eq. 3) is
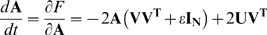
(A2)where **I**_N_ is the *N*×*N* identity matrix. **VV^T^** and **UV^T^** are both *N*×*N* matrices.

The optimum of *F* (Eq. A1) can be calculated from the equation of motion (Eq. A2) by setting the left hand side to zero and solving for **A**:
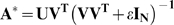
(A3)


Taking the limit 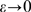 reduces Eq. (A3) to
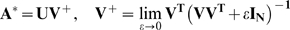
(A4)where **V^+^** is the pseudo-inverse of **V** satisfying 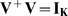. Using the fact that **U = MV** we obtain A^*^ = MVV^+^.

The solution in an MVG problem with *g* goals each with *k* input-output pairs can be calculated by taking the limit of vanishing small switching time. In this case the MVG problem is equivalent to the average problem
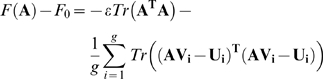
(A5)with the equation of motion
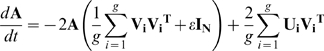
(A6)


Here we assume that equal amounts of time are spent in each goal. If this is not the case then the average over goals should be replaced by a weighted mean. Eq. (A6) can be further simplified by noting that
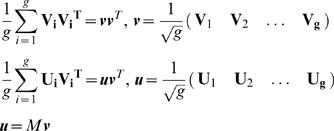
(A7)


Here 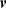, and 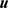 are both *N*×*(gk)* matrices in which all the input-output pairs are concatenated: the input vectors in a matrix 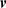, and the output vectors in a matrix 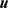.

With this, the equation of motion reads
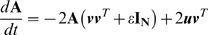
(A8)with the optimal solution
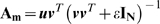
(A9)


We assume that *N* out of the *g*×*k* columns of 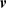 are linearly independent. Accordingly the rank of the rows is *N*. Thus 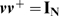 with 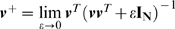 and so
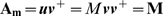
(A10)


The fitness, *F*, in the MVG problem is then
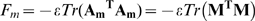
(A11)and similarly for a FG (fixed goal, in which the goal is constant over time) problem
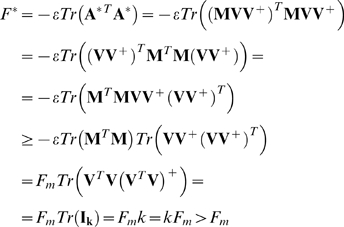
(A12)


Here we used the inequality 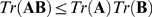. The conclusion is that F_m_<F^*^, that is the optimal fitness in an FG problem is higher than the fitness in an MVG problem.

#### (B) Eigenvalues of FG and MVG problems in *N* dimensional space

First we show that goals with *k* input-output pairs in *N* dimensions leads to evolutionary dynamics with *N−k* eigenvalues equal to *2ε*. Thus convergence (whose time goes as the inverse of the smallest eigenvalue) is slow. Then we show that in an MVG problem with *g* goals each with *k* input output pairs in *N* dimensions generically leads to evolutionary dynamics with effectively *no* eigenvalues equal to *2ε*. Thus convergence is faster.

We begin by writing the solution of Eq. (A2):
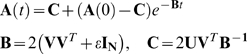
(B1)where **A**(0) is the initial condition. **B** is the coefficients matrix in Eq. (A2). Its eigensystem determine the dynamics described by Eq. (A2) and the solution (B1):
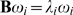
(B2)where 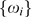 are the eigenvectors and 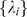 are their corresponding eigenvalues. The 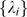 are the roots of the characteristic polynomial
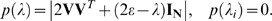
(B3)


We will show now that *N−k* of the roots of the characteristic polynomial *p*(*λ*) equal to 2*ε*. Using the formula for modified determinants 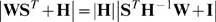 with 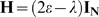, and 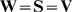, we can write:
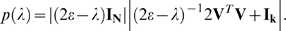
(B4)


Here *I_k_* and the *k*×*k* identity matrix, and **V** *^T^* **V** is a *k*×*k* Gram matrix – a symmetric semi-positive definite matrix whose eigenvalues are all nonnegative. Since we further assume that the columns of **V** are linearly independent then **V** *^T^* **V** is actually a positive definite matrix of rank *k*, whose eigenvalues are all positive. After factoring we find
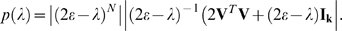
(B5)


Using the rule 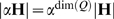 we find:
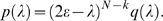
(B6)where 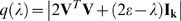 is a polynomial of degree *k*. In the limit 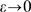, 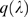 is the characteristic polynomial of the matrix 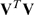, which is a full rank matrix. Thus, it has *k* non-vanishing eigenvalues. Accordingly the characteristic polynomial 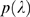 has *N−k* roots equal to *2ε* and *k* roots of *O(1)*.

Geometrically, this means that the dynamics in the *N* dimensional space can be separated into two regimes: fast dynamics on a *k* dimensional hyperplane (characterized by *k* large eigenvalues), and slow dynamics on the complementary *N−k* hyperplane (characterized by *N−k* eigenvalues equal each to *2ε*).

For completeness we write the solution (B1) in terms of the eigensystem of the coefficient matrix:
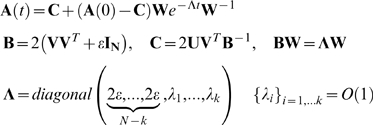
(B7)


Note that this solution holds for MVG problems. At the beginning of each epoch (after a goal switch) we update the initial conditions (equal to the value of the matrix **A** at the end of the previous epoch), and change the goal and corresponding eigensystem (update **V** and **U** for the next epoch).

Now we show that in an MVG problem with *g* goals each with *k* input-output pairs in *N* dimension generically leads to evolutionary dynamics with only large eignevalues, and *no* eigenvalues on the order of *ε*. Thus convergence is fast.

We approach this problem by taking the limit of vanishing small switching time. In this case the MVG problem is equivalent to the average problem with the equation of motion Eq. (A8). Thus the eigensystem in this case is determined by the characteristic polynomial of the average problem:
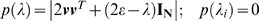
(B8)


In the generic case *N* out of the *g*×*k* columns of 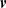 are linearly independent. Accordingly the rank of the rows is *N* and 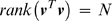. So that in the limit 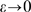, 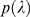 has *N* non-vanishing eigenvalues.

Geometrically, this means that unlike the dynamics in a FG problem, the dynamics in an MVG problem in *N* dimensional space is fast and generally characterized by *N* large eigenvalues. Note that if the epoch time is finite, then one can define a critical epoch time for which this result still holds (see Text S1).

For completeness we write the solution for the equation of motion (A8)
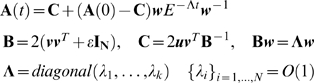
(B9)


## Discussion

We studied a model for evolution under temporally varying goals that can be exactly solved. This model captures some of the features previously observed with simulations of more complex systems [18],[19]: MVG leads to evolution of modular structures. The modules correspond to the correlations in the goals. Furthermore, evolution is speeded up under MVG relative to constant goals. The harder the goal is, the faster the speedup of MVG relative to evolution under a constant goal. Most random non-modular goals do not generally lead to speedup or evolution of modularity, but rather to evolutionary confusion. Although the modular solution is sub-optimal, it is selected for its ability to adapt to the different varying goals.

The speedup of evolution under MVG is a phenomenon that was previously found using simulations, but lacked an analytical understanding. The present model offers an analytical explanation for the speedup observed under MVG. The speedup in the model is related to small eigenvalues that correspond to motion along fitness plateaus when the goal is constant in time. These eigenvalues become large when the goal changes over time, because in MVG, the plateaus of one goal become a high-slope fitness region for the other goal. Switching between goals guides evolution along a ‘ramp’ that leads to the modular solution. This analytical solution of the dynamics agrees with the qualitative analysis based on sampling of the fitness landscape during the evolutionary simulations of complex models [19].

One limitation in comparing the present model to more complex simulations is that the present model lacks a complex fitness landscape with many plateaus and local maxima. Such plateaus and local fitness maxima make constant-goal evolution even more difficult, and are expected to further augment the speed of MVG relative to constant goal conditions. A second limitation of the present linear model is that it can solve different MVG goals when presented simultaneously - a feature not possible for nonlinear systems. This linearity of the model, however, provides a clue to how MVG evolution works: whereas each goal supplies only partial information, all goals together specify the unique modular solution. Under MVG evolution, the system effectively remembers previous goals, supplying the information needed to guide evolution to the modular solution, even though at each time point the current goal provides insufficient information. This memory effect is likely to occur in the nonlinear systems as well.

The series of studies on MVG, including the present theory, predict that organisms or molecules whose environment does not change over time should gradually lose their modular structure and approach a non-modular (but more optimal) structure. This suggestion was supported by a study that showed that bacteria that live in relatively constant niches such as obligate parasites that live inside cells, seem to have a less modular metabolic network than organisms in varying environments such as the soil [32],[33]. Another study considered modularity in proteins, which corresponds to distinct functional domains within the protein. It was found that proteins whose function is relatively constant over evolutionary time, such as the ribosomal proteins present in all cells, are typically less modular in structure than proteins that are specific to a few cell types and that repeatedly duplicate and specialize over evolution [34]. Thus, one might envisage a tradeoff in biological design between modularity and optimality. Modularity is favored by varying goals, and non-modular optimality tends to occur under more constant goals.

In summary, the present model provides an analytical explanation for the evolution of modular structures and for the speedup of evolution under MVG, previously found by means of simulations. In the present view, the modularity of evolved structures is an internal representation of the modularity found in the world [32]. The modularity in the environmental goals is learned by the evolving structures when conditions vary systematically (as opposed to randomly) over time. Conditions that vary, but which preserve the same modular correlations between inputs and outputs, promote the corresponding modules in the internal structure of the organism. The present model may be extended to study additional features of the interplay between spatio-temporal changes in environment and the design of evolved molecules and organisms.

## Supporting Information

Text S1 A Simple Model for Rapid Evolution of Modularity
(0.42 MB PDF)
