# Supplementary material for: An Analytically Solvable Model for Rapid Evolution of Modular Structure
Source: PLoS Comput Biol. 2009 Apr 10;5(4):e1000355. doi: 10.1371/journal.pcbi.1000355 (PMC2661363; doi:10.1371/journal.pcbi.1000355)
Supplement: Text S1 — A Simple Model for Rapid Evolution of Modularity (0.42 MB PDF) [file pcbi.1000355.s001.pdf]

An Analytically Solvable Model for Rapid Evolution of Modular Structure

Supporting Information

N. Kashtan*[1], A. E. Mayo*[1], T. Kalisky[1], U. Alon[1,2]

[1] Dept. of Molecular Cell Biology and [2] Physics of Complex Systems,
Weizmann Institute of Science, Rehovot Israel 76100

*These authors contributed equally to this work

The Supporting Information is organized as follows:

1. Modularity measure.

2. Analysis with Genetic Algorithms.

3. Speedup calculation.

4. The relation between the block structure of the evolved matrix and the covariance and correlation within the goal input and output vectors.

5. Calculation of the critical epoch time for speedup.

6. Other cost functions.

7. Solution under the assumption of no cost

8. Modularity declines if goals become constant

1. Modularity measure

In this section, we define the modularity measure used to quantify the modularity of the interaction network defined by the matrix **A**. An *NxN* matrix **A** can be mapped to a 2N-node network. In this mapping, the matrix element *A(i,j)* represents the weighted directed interaction between input-node *j* to output-node *(N+i)* (as described in Fig. S1).

To quantify network modularity we employed the measure based on the approach of Newman and Girvan [1,2], refined to weighted directed networks. Briefly, the Newman and Girvan algorithm finds the division of the nodes into modules that maximizes a measure Q. This measure is defined by the fraction of the edges in the network that connect between nodes in a module (or 'community' as termed by the authors) minus the expected value of the same quantity in a network

with the same assignment of nodes into modules but random connections between the nodes [3].

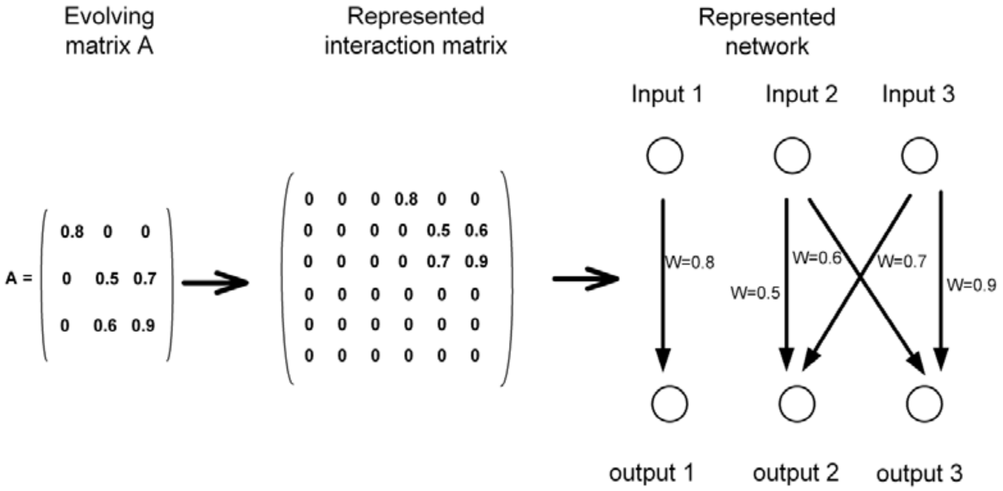

Figure S1. The *NxN* matrix **A** represents a weighted interaction network with *2N* nodes.

To measure modularity in a directed network where the edges have different weights we define the Q measure as follows:

(S1) $$Q = \sum_{s=1}^{K} \left[\frac{w_s}{W} - \left(\frac{d_s^{in} x d_s^{out}}{W^2} \right) \right]$$

where K is the number of modules, W is the total sum of the interaction weights in the network, w_s is the total sum of the interaction weights between nodes in module s, d_s^{in} is the sum of the weighted in-degrees of the nodes in module s and d_s^{out} is the sum of the weighted out-degrees of the nodes in module s. We assume all weights are non-negative, otherwise absolute values are taken.

We further refined this measure, as described in [4], by normalizing it with respect to randomized networks, (randomized matrices A of the same dimension *N)*. This is based on the observation that randomized networks typically do not show Q=0, due to the fact the even a random network has at least one partitioning of its nodes that yields a Q value well above zero. To address this, we used a normalized measure Q_m:

(S2) $Q_m=(Q_{real}-Q_{rand})/(Q_{max}-Q_{rand})$

where Q_{real} is the Q value of the network, Q_{rand} is defined as the average Q value of randomized networks, and Q_{max} is defined as the maximal possible Q value of the network.

To compute the modularity measure of a network, Q_m, we first calculated its Q_{real}. To measure Q_{rand} we used 10^4 random matrices. To estimate Q_{max} we used an upper bound $Q_{max}=1-1/K$ where K is the maximal number of modules, which can be considered here as N (the dimension of the matrix **A**).

2. Genetic Algorithm

The main text employed Hill-climbing dynamic to solve the evolution over time. Here, we describe results using a different evolutionary dynamics, employing a standard genetic algorithm (GA) [5-7] using the Matlab GA toolbox. In the genetic algorithm, the genome describes the values of the matrix A. We started with homogenous population initialized to a random A value with $|a_{ij}|<1$. Mutations were applied with probability $P_m=1$ per gene (matrix element) per generation. A mutation randomly changed elements in A by a shift drawn from the Gaussian 0 ± 0.005 (mean±SD). Population size was 500. Selection was fitness proportional, where the probability of passing to the next generation was proportional to fitness of each individual. Evolution time was defined as the number of generations required to reach distance $D(t) < D_0$ ($D_0=0.1$) from the desired goal (rms distance). We find that using genetic algorithms, there is a rapid convergence to the modular solution under MVG, and slow convergence to the optimal but non-modular solution under fixed goals (Figures S2-S4). Thus, we observe qualitatively similar results to the analytic results described in the paper.

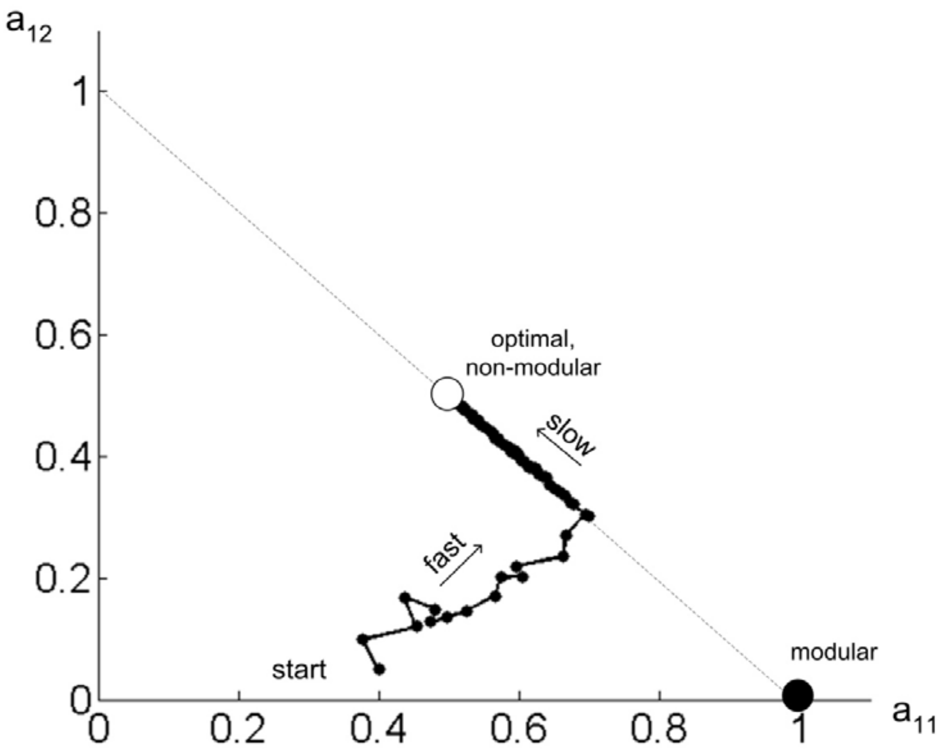

Figure S2. Evolutionary trajectories (using genetic algorithms) under fixed goal. a. A typical trajectory under fixed goal with G1=[v=(1,1), u=(1,1)] presented on the plane of the two matrix elements a_{11} and a_{22}. Black points represent best individual in the population, at a 20 generation temporal resolution. Empty circle: optimal non-modular solution (0.5,0.5); Full circle: modular solution (1,0). The cost parameter is ε=0.001.

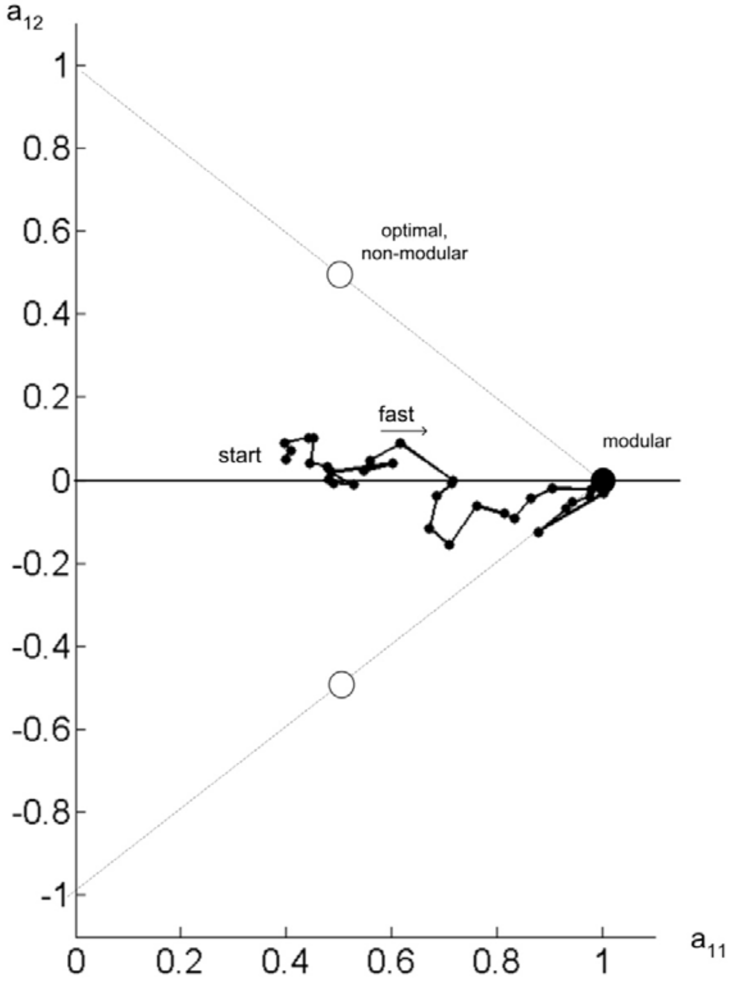

Figure S3. Evolutionary trajectories, using genetic algorithms, under MVG. A typical trajectory under MVG varying between G1=[v=(1,1), u=(1,1)] and G2=[v=(1,-1), u=(1,-1)] presented on the a_{11} and a_{22} plane. Black points represent best individual in the population, at a 20 generation resolution. Empty circle: optimal non-modular solutions (0.5,0.5) and (0.5,-0.5); Full circle: modular solution (1,0). Goals switched every E=100 generations. The cost parameter is ε=0.001.

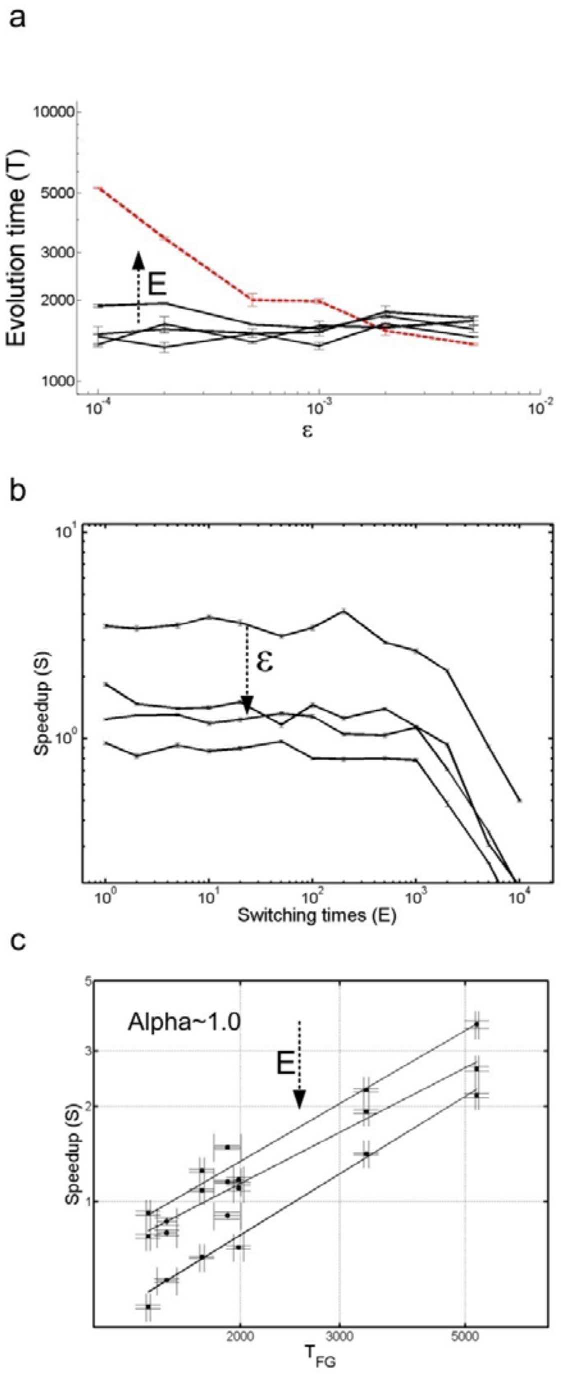

Figure S4. Speedup increases with problem difficulty, simulations using genetic algorithms. a. Time (no. of generations) to converge on the solution as a function of ε. Red dashed-line: under fixed goal evolution (T_{FG}), black: MVG with different goal switching times (E), increasing in the direction of the dashed arrow. (E=2,10,20,100). **b.** The Speedup S as a function of goal switching times (E). Lines are for different ε values, increasing in the direction of the dashed arrow (ε = 0.01,0.001,0.002,0.0001).**c.** Speedup as a function of T_{FG}. The speedup scales linearly with T_{FG} (as a power law $S \sim (T_{FG})^{\alpha}$ with an exponent α=1.0±0.1). Lines are for switching times (E=10,50,100), increasing in the direction of the dashed arrow.

3. Speedup Calculation

The speedup (S) is defined as the ratio of the time, starting from random initial conditions, to reach the solution in a fixed goal problem (T_{FG}) to the time the system reaches a solution in a modularly varying goal problem (T_{MVG}) :

$$S = \frac{T_{FG}}{T_{MVG}}$$

Note that the solution in an MVG problem is not a point but rather a limit cycle whose amplitude depends on the epoch time (E) and on the difficulty of the fixed goals composing the problem (ε). Accordingly, T_{MVG} was calculated as the time at which the system reaches its limit cycle. The amplitude of the limit cycle was also used to determine the threshold for the convergence of the system to its fixed goals (see Fig. S5).

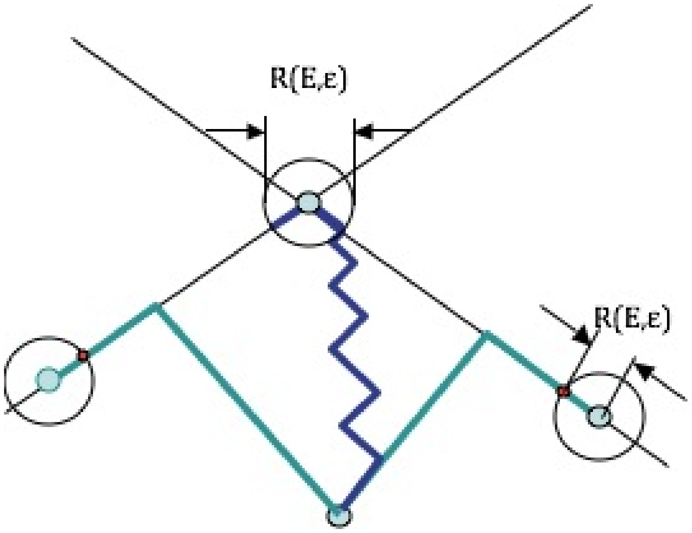

Figure S5. A schematic view of convergence under fixed goal (green lines) and under modularly varying goals (blue broken line). The amplitude of limit cycle is a function of epoch time (E) and η. T_{FG} is defined as the time it takes the system to reach the same distance from the optimal solution.

In case of *nearly* modularly varying goals, the definition above was modified to take into account the parameter characterizing the distance between the goals η (see Fig. S6). The limit cycle of the system was calculated, and the minimal distance

between the solution and the corresponding modular solution was found in each epoch. The threshold for calculation of T_{FG} was then taken as the mean of the two distances: R_m= Mean(R_1,R_2).

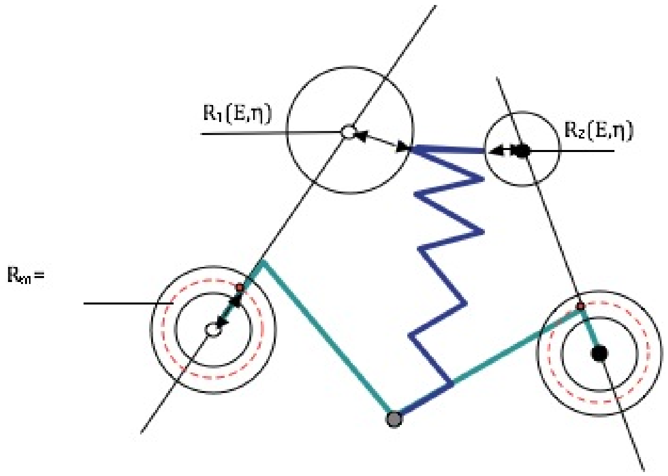

Figure S6. A schematic view of convergence under fixed goal (green lines) and under nearly modularly varying goals (blue broken line). The amplitude of limit cycle is a function of epoch time *E*) and η.

4. The block structure of the evolved matrix is determined by the correlation between the goal input and output.

Here we show the relation between the block structure of the evolved matrix (*A*) and the covariance structure of the goal input and output vectors (V and U). Here we will treat the inputs and outputs as *independent* random variables

$$V = (v_1, v_2, \ldots, v_N)$$
$$U = (u_1, u_2, \ldots, u_N)$$

By definition the goal *G* is modular if there exists a block diagonal matrix *M* such that *MV=U* (up to permutations of the columns of *V* and *U*). In terms of the v's and u's this reads:

$$u_i = \sum_{j=1}^{N} M_{ij} v_j \ .$$

Since each variable is sampled k times, the means are:

(S3)

$$\langle v_i \rangle = \frac{1}{k}\sum_{q=1}^{k}(v_i)_q$$

$$\langle u_i \rangle = \frac{1}{k}\sum_{q=1}^{k}(u_i)_q = \frac{1}{k}\sum_{q=1}^{k}\left(\sum_{j=1}^{N}M_{ij}v_j\right)_q = \frac{1}{k}\sum_{q=1}^{k}\sum_{j=1}^{N}M_{ij}(v_j)_q = \sum_{j=1}^{N}M_{ij}\frac{1}{k}\sum_{q=1}^{k}(v_j)_q = \sum_{j=1}^{N}M_{ij}\langle v_j \rangle$$

Here we used the linearity of the mean operator. Similarly, the second moments are:

(S4)

$$\langle v_i v_j \rangle = \frac{1}{k}\sum_{q=1}^{k}(v_i)_q(v_j)_q$$

$$\langle u_i v_j \rangle = \frac{1}{k}\sum_{q=1}^{k}(u_i)_q(v_j)_q = \frac{1}{k}\sum_{q=1}^{k}\left(\sum_{p=1}^{N}M_{ip}v_p\right)_q(v_j)_q = \frac{1}{k}\sum_{q=1}^{k}\left(\sum_{p=1}^{N}M_{ip}v_p\right)_q(v_j)_q =$$

$$= \frac{1}{k}\sum_{q=1}^{k}\sum_{p=1}^{N}M_{ip}(v_p)_q(v_j)_q = \sum_{p=1}^{N}M_{ip}\frac{1}{k}\sum_{q=1}^{k}(v_p)_q(v_j)_q = \sum_{p=1}^{N}M_{ip}\langle v_p v_j \rangle$$

$$\langle u_i u_j \rangle == \sum_{p=1}^{N}M_{ip}\langle v_p u_j \rangle = \sum_{p=1}^{N}M_{ip}\left(\sum_{n=1}^{N}M_{jn}\langle v_p v_n \rangle\right) = \sum_{n=1}^{N}\sum_{p=1}^{N}M_{ip}M_{jn}\langle v_p v_n \rangle$$

The covariance matrix of the goals input and output pairs can now be calculated:

(S5) $$C(u_i, v_j) = \langle u_i v_j \rangle - \langle u_i \rangle\langle v_j \rangle = \sum_{p=1}^{N}M_{ip}\left(\langle v_p v_j \rangle - \langle v_p \rangle\langle v_j \rangle\right) = \sum_{p=1}^{N}M_{ip}C(v_p, v_j)$$

Note that like M, the covariance matrix is an *NxN* matrix. Since the *v*'s are independent variables, $C(v_p, v_j) = \sigma(v_j)^2\delta_{pj}$. Substituting this into Eq. (S5), doing the summation, and solving for M_{ij} we find:

(S6) $$M_{ij} = \frac{C(u_i, v_j)}{\sigma(v_j)^2}$$

Writing the covariance matrix in terms of the correlation matrix

$$C(u_i, v_j) = \rho(u_i, v_j)\sigma(u_i)\sigma(v_j),$$

we get

(S7) $$\rho(u_i, v_j)\sigma(u_i) = M_{ij}\sigma(v_j).$$

This can be further simplified by calculating $\sigma(u_i)$:

$$\sigma(u_i)^2 = C(u_i, u_i) = \sum_{p=1}^{N}M_{ip}C(v_p, u_i) = \sum_{p=1}^{N}M_{ip}C(u_i, v_p) = \sum_{p=1}^{N}M_{ip}M_{ip}\sigma(v_p)^2 = \sum_{p=1}^{N}M_{ip}{}^2\sigma(v_p)^2$$

Substituting this into (S7) we get

(S8) $$M_{ij} = \rho(u_i, v_j)\sqrt{\sum_{p=1}^{N}M_{ip}{}^2\frac{\sigma(v_p)^2}{\sigma(v_j)^2}}$$

Without loss of generality we can assume that the inputs are set to have zero mean and unit standard deviation, namely

(S9) $$\sigma(v_p) = \sigma(v_j)^2 = 1$$

Accordingly, if we define

(S10) $\widetilde{M}_{ij} \equiv \frac{M_{ij}}{\sqrt{\sum_{p=1}^{N} M_{ip}^{2}}}$,

That is the matrix M normalized so that the norm of the rows is 1, we finally obtain

(S11) $\widetilde{M}_{ij} = \rho(u_i, v_j)$.

Note that the structures of M and that $\widetilde{M}_{ij}$ are the same. Now based on appendix A (of the main text) we know that under MVG, the matrix A evolves towards the matrix M. And so it must evolve towards the structure of the correlation matrix between the input and output pairs.

5. Calculation of the critical epoch time for speedup.

In this section, we comment on the range of goal switching times where speedup can be observed in MVG relative to fixed goal problems. Following Eq. (5) in the main text, the evolution of the components $a_{ij}(t)$ of the mapping a_{ij}^* matrix **A** within a single epoch can be described by the following equation:

$$\text{(S12)} \quad a_{ij}(t) = \sum_{n}^{N} K_n e^{-\lambda_n t} + a_{ij}^*$$

Where a_{ij}^* is the optimal solution and the prefactors $\{K_n\}$ are determined by the eigenvectors corresponding to $\{\lambda_n\}$ and the initial conditions in that particular epoch. Taking the case of N=2 we can write:

$$\text{(S13)} \quad r(t) = a_{ij}(t) - a_{ij}^* = K_1 e^{-\lambda_1 t} + K_2 e^{-\lambda_2 t}$$

Where λ_1 is the large eigenvalue and λ_2 is the small one. The evolution can thus be decomposed into a fast and a slow component corresponding to the large and small eigenvalues respectively. Generally, at the beginning of the epoch the first component decays faster and governs the dynamics. However, at a certain switching rate the first component will become arbitrarily small and the second slowly decaying component will be dominant. It is beyond this point that speedup ceases.

Formally, for rapid evolution to occur, the *rate* of the rapid decay must be larger than the rate of the slow decay in each epoch. Hence:

$$\text{(S14)}\quad \left|\frac{d}{dt}\left(K_1 e^{-\lambda_1 t}\right)\right| > \left|\frac{d}{dt}\left(K_2 e^{-\lambda_2 t}\right)\right|$$

Or:

$$\text{(S15)}\quad \lambda_1 K_1 e^{-\lambda_1 t} > \lambda_2 K_2 e^{-\lambda_2 t}$$

$$e^{(\lambda_1 - \lambda_2)t} < \frac{\lambda_1 K_1}{\lambda_2 K_2}$$

$$t < \frac{1}{\lambda_1 - \lambda_2} \log\left(\frac{\lambda_1 K_1}{\lambda_2 K_2}\right)$$

Thus a general condition for speedup is that the epoch time will be less than some a epoch time:

$$\text{(S16)}\quad E_c = \frac{1}{\lambda_1 - \lambda_2} \log\left(F\left(\lambda_1, \lambda_2, K_1, K_2\right)\right)$$

Where $F(\;)$ is a function of the initial conditions (and the eigenvalues) upon which T_c depends weakly.

For epoch times obeying $T < T_c$ the dynamics is governed by the rapid decay component only (see Fig. S4 b). Hence, as long as $T < T_c$ the evolution time is essentially the same. However, if the epoch time increases beyond T_c the added time will be spent along the slow component, and total evolution time will increase with the added time being linearly proportional to $T - T_c$.

6. Other cost functions

As mentioned in the main text, one can test various cost functions (the benefit function is maintained as the Frobenius norm since we measure the Euclidian distance from the goal). The cost function we presented in the main text has the advantage of a fully solvable linear dynamics. To study the dynamics governed by other cost we used two different methods: numerical analysis and simulations using genetic algorithms. We find qualitatively similar results and dynamics to those found in the main text, under a wide range of convex cost functions. Concave cost functions can lead to scenarios in which the modular solution is in fact the optimal solution (either local or global). Thus it can be found in FG problems as well as in MVG problems. However, it is not guaranteed that the system will actually reach the modular solution since it may be flanked by other local optima. This is avoided in MVG problems. The non-convex cost |a| is of special interest since it is characterized by a true fitness plateau.

To study a broader range of cost functions, we examined a family of cost functions such as:

$$\text{(S17)}\quad c = \varepsilon \sum_{ij} \frac{|a_{ij}|^n}{K^n + |a_{ij}|^n}$$

where n > 0 and $K \cong 1$. For n>1 these cost functions are convex (concave) for $K > |a_{ij}|$ ($K < |a_{ij}|$), and strictly concave for 0 < n < 1.

Specifically, in the limit $K >> |a_{ij}|$ the cost function (S17) reduces to $c = \varepsilon K^{-n} \sum_{ij} |a_{ij}|^n$ which upon the redefinition $\varepsilon \to \varepsilon K^n$ and n=2 becomes the cost function of the main text. Now, although the dynamic is affected by the new cost function, the result that in a MVG problem the obtained solution is the modular one still remains. Here we show this analytically in a 2D case by calculating the equations of motion described by the modified fitness function, and looking for the equilibrium points.

In the simple 2D FG problem we get for the first row of the matrix A

$$F = -\varepsilon\left(|a_{11}|^n + |a_{12}|^n\right) - \left(v_{11}a_{11} + v_{12}a_{12} - u_{11}\right)^2 .$$

Assuming that the system is allowed to reach the low gradient ridge, and is now constrained to move along it (that is the benefit is maximal) the matrix elements are related by:

$$v_{11}a_{11} + v_{12}a_{12} - u_{11} = 0 .$$

Solving for a_{12}, and substituting back into F we get up to correction of $O(\varepsilon^2)$, the cost along the ridge:

(S18) $$F = -\varepsilon\left(\left|a_{11}\right|^n + \left|\frac{v_{11}}{v_{12}}\right|^n \left|\frac{u_{11}}{v_{11}} - a_{11}\right|^n\right).$$

Differentiating F with respect to a_{11} we can find the equation for the equilibrium:

(S19) $$Sign(a_{11})\left|a_{11}\right|^{n-1} = Sign\left(\frac{u_{11}}{v_{11}} - a_{11}\right)\left|\frac{v_{11}}{v_{12}}\right|^n \left|\frac{u_{11}}{v_{11}} - a_{11}\right|^{n-1}.$$

From this it follows that the solution must obey $Sign(a_{11}) = Sign(u_{11}/v_{11} - a_{11})$ (or to put it differently, depending on the sign of u_{11}/v_{11}, $0 < a_{11} < u_{11}/v_{11}$ or $u_{11}/v_{11} < a_{11} < 0$). Thus up to corrections of $O(\varepsilon)$, the only solution is

(S20) $$a_{11} = \frac{u_{11}}{v_{11}} \frac{1}{\left|v_{11}/v_{12}\right|^{n/(1-n)} + 1}.$$

Note that if $u_{11} = v_{11} = v_{12} = 1$, we recover the solution of the main text: $a_{11}=1/2$ for all $n \neq 1$. Calculating the second derivative shows that solution (S20) is a minimum for n > 1 (convex cost function). For 0 < n < 1 (concave cost function) this solution is a local maximum flanked by two minima. F is not differentiable there nevertheless the solution can be found: for $v_{12} < v_{11}$ ($v_{12} > v_{11}$) we have one local (global) maximum at $a_{11} = 0$ and one global (local) maximum at $a_{11} = \frac{u_{11}}{v_{11}}$. This means that for 0 < n < 1 the modular solution ($u_{11} = v_{11}$) is in fact a maximum (either local or global). Thus, for concave cost functions, modularity can be obtained in a FG problem as well as in a MVG problem.

In the special case of $n=1$, F is not differentiable. Nevertheless the minimum can still be found. It is obtained at $a_{11} = \frac{u_{11}}{v_{11}}$ for $v_{12} \neq v_{11}$. Interestingly, if $v_{12} = v_{11}$ the minimum is obtained at any point in the closed section $[0, u_{11}/v_{11}]$. That is the fitness landscape has a true fitness plateau.

7. Solution under the assumption of no cost

Here we will derive the solution of a FG problem assuming that there is no cost. We begin with the general solution (B6) in the limit of ε=0:

$$A(t) = C + (A(0) - C)We^{-\Lambda t}W^{-1}$$
$$B = 2VV^T, \quad C = 2UV^T B^{-1} \quad BW = \Lambda W$$
$$\Lambda = diagonal\left(\underbrace{0,\ldots,0}_{N-k},\lambda_1,\ldots\lambda_k\right)$$

Accordingly in the steady state solution

$$e^{-\Lambda t} = diagonal\left(\underbrace{1,\ldots,1}_{N-k},e^{-\lambda_1 t},\ldots e^{-\lambda_k t}\right)\xrightarrow{t\to\infty}\begin{pmatrix} I_{N-k} & 0 \\ 0 & 0\end{pmatrix}$$

Where $I_{N\text{-}k}$ is the N-k dimensional identity matrix.

$$\lim_{t\to\infty} A(t) = C + (A(0) - C)W\begin{pmatrix} I_{N-k} & 0 \\ 0 & 0\end{pmatrix}W^{-1} =$$
$$= C + (A(0) - C)W\left(I_N - \begin{pmatrix} 0 & 0 \\ 0 & I_k\end{pmatrix}\right)W^{-1} =$$
$$= A(0)\left(I_N - W\begin{pmatrix} 0 & 0 \\ 0 & I_k\end{pmatrix}W^{-1}\right) + CW\begin{pmatrix} 0 & 0 \\ 0 & I_k\end{pmatrix}W^{-1}$$

In order to proceed we make use of the fact that $\begin{pmatrix} 0 & 0 \\ 0 & I_k\end{pmatrix} = \Lambda\Lambda^+$:

$$W\begin{pmatrix} 0 & 0 \\ 0 & I_k\end{pmatrix}W^{-1} = W(\Lambda\Lambda^+)W^{-1} = (W\Lambda W^{-1})(W\Lambda^+ W^{-1}) = (W\Lambda W^{-1})(W\Lambda W^{-1})^+ = BB^+$$

Using the definition of C we obtain:

$$CBB^+ = (2UV^T B^+)BB^+ = 2UV^T(B^+BB^+) = 2UV^T B^+ = C$$

Substituting this back we finally get:

$$\lim_{t\to\infty} A(t) = A(0)(I_N - BB^+) + C$$

Assuming now that the initial conditions for the problem, A(0) are distributed uniformly in $R^{N\times N}$ means that $\langle A(0)\rangle = 0$. Accordingly, the equilibrium solution averaged over all initial conditions is:

$$\lim_{t\to\infty}\langle A(t)\rangle = \langle A(0)(I_N - BB^+) + C\rangle = \langle A(0)\rangle(I_N - BB^+) + \mathrm{C} = \mathrm{C}$$

Thus we finally get $\lim_{t\to\infty}\langle A(t)\rangle = C$ which is the optimal solution with non-vanishing cost.

8. Modularity declines if goals become constant:

What happens to modularity under a constant goal if one begins with a modular solution as an initial condition? We find that modularity decays over time

(Fig 8b). Generally, this decay corresponds to motion along the low gradient valley towards the optimal, non-modular fixed point. Hence the typical time constant is of order 1/ε. In the relatively simple two-dimensional case this can be shown analytically. Here the modularity measure Q (see supporting information for definition) is

$$Q_{D=2} \propto Det(|\mathbf{A}|) \Big/ \left(\sum_{ij} |a_{ij}| \right)^2 .$$

Now, since $\mathbf{A}$ converges to the optimal solution $\mathbf{A}^*$ (which is rank deficient), $Det(|\mathbf{A}|) \to Det(|\mathbf{A}^*|) = 0$ in the limit $t \to \infty$, and so $Q_{D=2} \to 0$ also in this limit. Furthermore, $Q_{D=2}$ can be calculated analytically by using the solution to the equations of motions as given by eq. (5) and using the fact that the modular initial conditions puts the system inside the valley, which means that fast terms in $\mathbf{A}$ can be neglected (expressions that depends on the large eigenvalue λ). Substituting into $Q_{D=2}$ we get:

$$Q_{D=2} \propto \frac{\left|(a_{11}^* + h_{11}E^{-\varepsilon t})(a_{22}^* + h_{22}E^{-\varepsilon t})\right| - \left|(a_{12}^* + h_{12}E^{-\varepsilon t})(a_{21}^* + h_{21}E^{-\varepsilon t})\right|}{\left(\sum \left|a_{ij}^* + h_{ij}E^{-\varepsilon t}\right|\right)^2} + O\left(E^{-\lambda t}\right)$$

where a_{ij}^* are the components of the optimal solution, and h_{ij} are constants determined by the eigensystem of the problem. Expanding while keeping terms of order $E^{-\varepsilon t}$, and using the fact that $Det(|A^*|) = a_{11}^* a_{22}^* = a_{12}^* a_{21}^* = 0$, we finally get

$$Q_{D=2} \propto \frac{\left|a_{11}^* a_{22}^*\right|}{\left(\sum \left|a_{ij}^* + E^{-\varepsilon t} h_{ij}\right|\right)^2} \left(\left|1 + w_1 E^{-\varepsilon t}\right| - \left|1 - w_2 E^{-\varepsilon t}\right| \right) .$$

where w_1 and w_2 are constants. The result is that $Q_{D=2}$ vanishes exponentially slowly ($\sim E^{-\varepsilon t}$). Note that since Q_m is a linear function of Q (see supporting information), these results still hold for Q_m.

The conclusion is that if left alone, the modularity of the system decays slowly to the modularity of the optimal solution. Thus, goals need to keep varying over time in order to maintain the modular structure.

References

1. Newman MEJ, Girvan M (2004) Finding and evaluating community structure in networks. Phys Rev E 69: 026113.
2. Newman MEJ (2004) Fast algorithm for detecting community structure in networks. Phys Rev E 69: 066133.
3. Guimera R, Nunes Amaral LA (2005) Functional cartography of complex metabolic networks. Nature 433: 895-900.
4. Kashtan N, Alon U (2005) Spontaneous evolution of modularity and network motifs. Proc Natl Acad Sci U S A 102: 13773-13778.
5. Goldberg D (1989) Genetic Algorithms in Search, Optimization, and Machine Learning: Addison-Wesley Publishing Company, Inc.
6. Holland J (1975) Adaptation in natural and artificial systems: The University of Michigan Press.
7. Mitchell M (1996) An Introduction to Genetic Algorithms. Cambridge MA: MIT Press.
